# Early Cerebrovascular Autoregulation in Neonates with Congenital Heart Disease

**DOI:** 10.3390/children9111686

**Published:** 2022-11-03

**Authors:** Celina L. Brunsch, Mirthe J. Mebius, Rolf M. F. Berger, Arend F. Bos, Elisabeth M. W. Kooi

**Affiliations:** 1 Neonatology, Beatrix Children’s Hospital, University Medical Center Groningen, University of Groningen, 9713GZ Groningen, The Netherlands; 2 Center for Congenital Heart Disease, Pediatric Cardiology, Beatrix Children’s Hospital, University Medical Center Groningen, University of Groningen, 9713GZ Groningen, The Netherlands

**Keywords:** neonatology, congenital heart disease, cerebrovascular circulation, brain injury, hemodynamics, arterial pressure

## Abstract

Neonates with congenital heart disease (CHD) display delayed brain development, predisposing them to impaired cerebrovascular autoregulation (CAR) and ischemic brain injury. For this paper, we analyzed the percentage of time with impaired CAR (%time impaired CAR) during the first 72 h after birth, the relation with clinical factors, and survival in 57 neonates with CHD. The primary outcome was a correlation coefficient of cerebral oxygenation (r_c_SO_2_) and mean arterial blood pressure (MABP, mmHg) for two hours on a daily basis. The %time impaired CAR ranged from 9.3% of the studied time on day one to 4.6% on day three. Variables associated with more %time impaired CAR were the use of inotropes (day 1, B = 19.5, 95%CI = 10.6–28.3; day 3, B = 11.5, 95%CI = 7.1–16), lower MABP (day 1, B = −0.6, 95%CI = −1.2–0.0), and dextro-transposition of the great arteries (dTGA) (16.2%) compared with other CHD types (2.0–5.0%; day 1, *p* = 0.022). Survival was not an associated variable. To summarize, impaired CAR was found in CHD neonates in up to 9.3% of the studied time. More evidence is necessary to evaluate an association with inotropes, dTGA, %time impaired CAR, and long-term outcome, further in larger cohorts.

## 1. Introduction

Treatment of congenital heart disease (CHD) has improved over the past decades, which has resulted in lower mortality and an increase in the prevalence to 3000 per million [1,2,3]. Therefore, the focus has turned towards the prevention of brain injury and improvement of neurological outcomes in severe CHD [3,4,5,6]. The etiology of impaired neurological outcome in CHD is most likely multifactorial, and may already start before neonatal cardiac surgery [6], and even before birth [7,8]. Both delayed brain development and circulatory alterations with impaired oxygen supply inducing hypoxic brain injury may be involved [7,8,9,10,11,12].

In fact, delayed or disturbed brain development could also lead to impaired cerebrovascular autoregulation (CAR), which in turn may cause harmful cerebral blood flow (CBF) fluctuations. Under healthy circumstances, CAR maintains a constant CBF regardless of changing cerebral perfusion pressures (CPP) [13]. Beyond the autoregulatory range, defined by individual upper and lower CPP thresholds, passive collapse or passive dilation of cerebral blood vessels may lead to ischemia or hemorrhage [14,15]. A lower gestational age (GA) or birth weight (BW), higher arterial PCO_2_, and brain immaturity are known to influence the autoregulatory range and potentially lead to impaired CAR [16,17].

Premature infants are at high risk of CAR impairments, which may be caused by the immaturity of the cerebral vasculature and a narrower autoregulatory range [13,14,18]. Impaired CAR may lead to impaired cerebral oxygen supply, which has been linked with ischemic brain injuries such as periventricular echodensities (PVEs) preceding periventricular leukomalacia [19], and impaired neurological outcomes in premature infants [18]. Since current literature points towards delayed brain maturation in neonates with CHD, impaired CAR and abnormal neurological outcomes may also be found in these neonates [5,6,9,10,20]. Furthermore, neonates with CHD may be more prone to alternating CPP due to circulatory disturbances. Such disturbances include impaired cardiac function and intra- or extracardiac shunts affecting blood flow to the brain, potentially increasing the chance of CPP outside the autoregulatory range [14]. This is likely to be dependent on the type of CHD.

Since it is not possible to directly measure CPP and CBF continuously, mean arterial blood pressure (MABP) and cerebral oxygen saturation (r_c_SO_2_) using near-infrared spectroscopy (NIRS) have become accepted surrogate measures [13,14,16,21]. NIRS enables continuous, non-invasive bedside monitoring of r_c_SO_2_ and is standard practice in neonates with CHD at the University Medical Center Groningen (UMCG) [21]. To date, Votava-Smith et al. are the only group that investigated CAR continuously in neonates with CHD [22]. They found impaired CAR during 15.3% of the studied time; however, they studied a small population (24 neonates) without follow-up to assess the outcome.

The aim of this study was to evaluate the percentage of time with impaired CAR (%time impaired CAR) in neonates with CHD during the first 72 h after birth, which is the period of interest in this study due to the transitional changes taking place at this time [16]. Second, we assessed which clinical factors were associated with the %time impaired CAR. We also assessed the association between %time impaired CAR and early signs of hypoxia/ischemia on cerebral ultrasound, and survival. We hypothesized that %time impaired CAR would be approximately 15% of the time studied in neonates with severe CHD [22]. We expected a higher %time impaired CAR with CHD types such as dextro-transposition of the great arteries (dTGA) and hypoplastic left heart syndrome, due to intrauterine cerebral hypoxemia possibly associated with delayed brain development [7,8,23].

## 2. Materials and Methods

### 2.1. Patients and Data Collection

We conducted a retrospective analysis at the neonatal intensive care unit (NICU) of the Beatrix Children’s Hospital at the UMCG.

We included all term neonates with severe CHD who were admitted to the NICU between March 2015 and September 2020, when measuring r_c_SO_2_ using NIRS in infants with CHD had become standard care. We defined severe CHD as requiring admission to the NICU for hemodynamic support and due to anticipated ductal dependency. Inclusion criteria were: available r_c_SO_2_ and continuous MABP measurements via an indwelling arterial catheter during the first 72 h after birth. We excluded neonates with a GA < 36 weeks or major chromosomal abnormalities. The medical ethical committee of the UMCG permitted the execution of this study as it was regarded as a retrospective evaluation of standard care, which according to the Dutch Medical Law does not require parental consent (RR202000618).

### 2.2. Time with Impaired Cerebrovascular Autoregulation

We determined the %time impaired CAR in neonates with severe CHD during two-h monitoring episodes in the first 72 h after birth. CAR can be defined as the correlation between CBF and CPP. We used the MABP and r_c_SO_2_, measured with NIRS, as surrogates for CPP and CBF [16]. For r_c_SO_2_ measurements, we used the INVOS 5100 spectrometer (Medtronic, Dublin, Ireland) with neonatal sensors (Medtronic, Dublin, Ireland). MABP was measured invasively with a pressure transducer using an indwelling arterial catheter. Both measurements, and SpO_2_, are automatically transferred to a secure database and stored offline with a sampling frequency of 0.2 Hz.

We calculated correlation coefficients between MABP and r_c_SO_2_ using a moving window of 10 min and maximal overlap for every paired sample [16,22,24]. We calculated %time impaired CAR for 2 h of valid data (well-documented, correct sensor position and stable) per 24-h-period [25,26,27]. More specifically, we selected the CAR assessment periods based on several criteria: We selected an episode of continuously available MABP and r_c_SO_2_ values, after documenting the appropriate sensor position, according to the local NIRS protocol, preferably during the morning after nursing care, to avoid bias by variations due to a potential circadian rhythm. We allowed for a maximum of 30 s of missing values during the 2 h. We allowed fluctuations in both values but manually removed obvious artifacts (i.e., values of 0 or >100 for MABP, r_c_SO_2_, or SpO_2_). We yielded 1320 correlation coefficients for each 2 h measurement period, per day, per neonate. We defined impaired CAR as the correlation coefficient between r_c_SO_2_ and MABP, being above a cutoff of 0.3 as reported in previous studies [28,29,30]. A recent literature review reported cutoffs ranging between the values 0–0.5 [14] as is reflected in a range of different studies [26,27,28,29,30,31,32]. International consensus on a cutoff for impaired CAR is lacking, therefore we decided to choose this cutoff of 0.3, which is within the reported range of 0–0.5, but higher than 0 to compensate for potential measurement imprecisions.

We collected clinical data, including the CHD type, GA, BW, 5 min Apgar score, prostaglandin E_1_, sedatives, diuretics, inotropes, mechanical ventilation, persistent pulmonary hypertension of the newborn (PPHN), sepsis, blood transfusions, and PCO_2_ as possible determinants of CAR. We used PCO_2_ values that were determined <5 h before or after the CAR assessment period for neonates without invasive ventilation, or <2 h before or after the CAR assessment period for neonates with invasive ventilation. We defined signs of cerebral ischemia/hypoxia as PVE on transcranial Doppler ultrasound within two weeks after birth, before corrective surgery [19]. Transcranial Doppler ultrasound was performed through the anterior fontanel as soon as possible after birth in this population and repeated upon indication. In neonates with dTGA, we noted the balloon atrial septostomy as a potential variable associated with %time impaired CAR. Lastly, we recorded the survival of the neonates during the first month after birth [24,33].

### 2.3. Statistical Analysis

We performed statistical analyses using SPSS 23 software (IBM SPSS Statistics, IBM Corp., Armonk, NY, USA). Our primary outcome was reported as the %time impaired CAR during 2 h of measurement per day.

To assess the association between clinical data and the %time impaired CAR, we first performed a univariate regression. For the multivariable analysis (Model 1), we simultaneously entered all variables with *p* < 0.1 from the univariable analysis. We composed a second multivariable model (Model 2) with MABP as an additional variable. We performed a *t*-test to test MABP levels between infants with and without inotropes, as persistent low MABP despite inotrope administration may lead to CPP outside of the autoregulatory plateau. We compared PVE and survival with %time impaired CAR using a Mann–Whitney U test. For all statistical tests, we considered *p* < 0.05 to be statistically significant.

## 3. Results

### 3.1. Patient Characteristics

**Figure 1:**
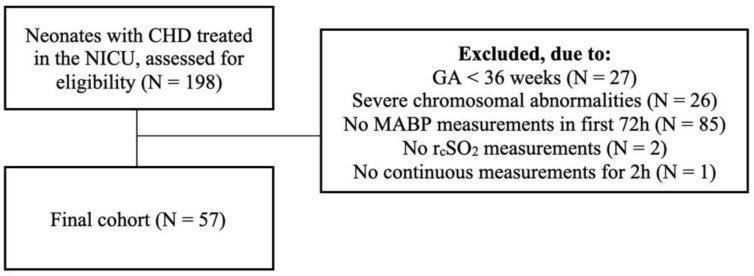
Inclusion diagram. Of the 198 neonates treated in our neonatal intensive care unit, 57 met the inclusion criteria for this study.

Between March 2015 and September 2020, 198 neonates with CHD were treated in our NICU, of whom 57 were included (Figure 1).

The main CHD type was dTGA in 24 neonates (42%). We classified the remaining defects as right-sided obstructive lesions, hypoplastic aortic arch or coarctation, and other defects (Supplemental Digital Table S1). Of all neonates with dTGA, 14 (58%) had undergone a balloon atrial septostomy within the first week after birth. In one of these, this was attempted but unsuccessful. Three non-dTGA neonates received cardiac procedures, of which two had a balloon atrial septostomy (one with pulmonary stenosis and one with aortic stenosis), and one with total anomalous pulmonary venous return who received surgical correction on day 2. In 17 (30%) and 11 (19%) of the 57 neonates, the pulmonary or systemic circulation was considered duct-dependent, respectively. Inotropes (dobutamine, dopamine, noradrenaline, milrinone, or a combination) were given to up to 24% of the neonates (Table 1). The median (range) dosage was 7.5 (3.25–10.0) µg/kg/min for dopamine, 5 (5.0–15.0) µg/kg/min for dobutamine, 0.5 (0.25–0.75) µg/kg/min for milrinone, and 0.08 (0.05–0.15) µg/kg/min for noradrenaline. The most frequently used inotropic medication was dopamine (day 1 = 90%, day 2 = 69%, day 3 = 70%). From the neonates with a need for intubation, 23 were intubated due to the underlying CHD, 5 after receiving prostaglandin E_1_ and apnea, 3 because of PPHN, 2 during balloon atrial septostomy, and 1 due to respiratory insufficiency not otherwise specified.

### 3.2. Time with Impaired Cerebrovascular Autoregulation

For all neonates, the median %time impaired CAR during the 2 h monitoring episode was 9.3% on day 1, 5.7% on day 2 and 4.6% on day 3 (Table 2).

### 3.3. Determinants of Time with Impaired CAR

**Figure 2:**
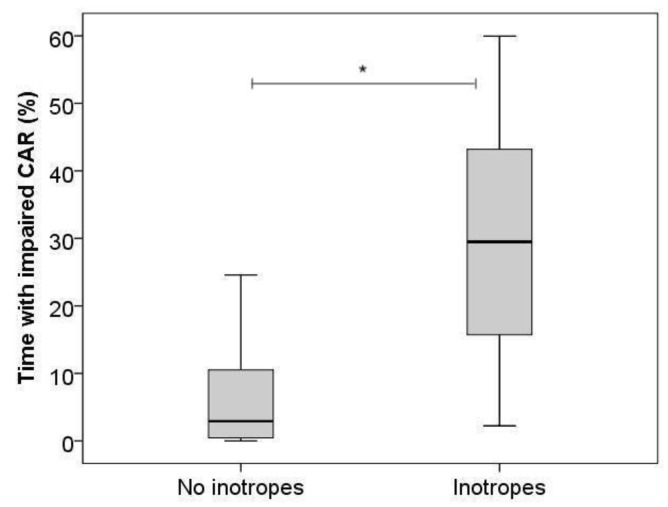
Boxplot, percentage of time with impaired cerebrovascular autoregulation on day 1 with and without the use of inotropes. Horizontal bars show medians, boxes show upper and lower quartiles, and whiskers show range. * indicates *p*-value < 0.05.

On day 1, we found significantly higher %time impaired CAR with the use of inotropes (B = 20.5, 95%CI [11.3–29.6]) for Model 1 (Figure 2), and with both inotropes (B = 19.5, 95%CI [10.6–28.3]) and lower MABP (B = −0.6, 95%CI [−1.2–0.0]) for Model 2.

On day 2, no variables were significantly associated with higher %time impaired CAR upon multivariable analysis (Supplemental Digital Table S2).

On day 3, only inotropes were associated with higher %time impaired CAR (B = 11.5, 95%CI [7.1–16.0]) (Supplemental Digital Table S3).

We found a significantly lower median (range) MABP for neonates who received inotropes (42.2 [33.3–49.4]), versus neonates without inotropes (45.8 [33.5–60.7]) on day 1 (*p* = 0.049), on day 2 (45.5 [36.9–63.6]; 47.8 [41.2–62.5]; *p* = 0.031), and on day 3 (44.3 [39.7–51.1]; 49.6 [38.9–61.7]; *p* = 0.015), respectively.

Neonates with dTGA had a significantly higher median %time impaired CAR on day 1 (16.2%), compared with every other CHD group (right-sided = 2.0%, hypoplastic aortic arch/coarctation 2.4%, other = 5.0%; *p* = 0.022). On days 2 and 3, this difference was no longer significant. Between 28.6–30.8% of the dTGA neonates received inotropic medication, compared with 7.1–12.5% for right-sided lesions, 10.0–12.5% for hypoplastic aortic arch or coarctation of the aorta, and 0.0–40.0% for other CHD types (Supplemental Digital Table S4).

### 3.4. PVE and Mortality

We found PVE in 11 (19%) of the studied neonates. We did not find a significant difference in the median %time impaired CAR between neonates with (day 1 = 10.0%, day 2 = 2.9%, day 3 = 5.3%) and without (day 1 = 9.2%, day 2 = 6.8%, day 3 = 4.5%) PVE (day 1 *p* = 0.65, day 2 *p* = 0.20, day 3 *p* = 0.99).

In total, 50 patients (88%) survived until the end of this study with a follow-up time of one month. Patients who did not survive died at a median (range) age of 18 (9–21) days. We did not find a significant difference in the median %time impaired CAR between survivors (day 1 = 6.2%, day 2 = 5.6%, day 3 = 4.9%) and non-survivors (day 1 = 15.7%, day 2 = 9.2%, day 3 = 3.3%) (day 1 *p* = 0.31, day 2 *p* = 0.43, day 3 *p* = 0.94).

## 4. Discussion

In this study, we found impaired CAR during 4.6–9.3% of the daily 2 h monitoring episodes in neonates with CHD within the first 72 h after birth. The administration of inotropes, as well as lower MABP, were associated with higher %time impaired CAR. A diagnosis of dTGA was also associated with more %time impaired CAR. We could not demonstrate a relation between %time impaired CAR and PVE or survival.

The %time impaired CAR in this study was lower than the values reported by others. Votava-Smith et al. found a %time impaired CAR of 15.3% in preoperative neonates with CHD. Their study population consisted of 58% left-sided lesions, 33% right-sided lesions and 8% TGA [22]. Additionally, 67% of their cohort were single ventricles, compared with a smaller number in our study, which we did not report specifically as a separate group. We propose two explanations: Firstly, their cutoff (0.58) of significant coherence between MABP and r_c_SO_2_ was based on a mathematical approach described by Taylor et al. [34]. Importantly, Votava-Smith et al. and Taylor et al. assessed CAR in the frequency domain, using coherence analysis, which is a different method to relate MABP changes to simultaneous r_c_SO_2_ measurements. Taylor et al. assessed RR-interval oscillations in healthy adults, using ECG and MABP recordings in a moving-window correlation analysis. Their mathematical approach to define a cutoff for this correlation was based on an F-test using window length and sampling frequency. Both Taylor et al. [34] and Votava-Smith et al. applied 10 min windows and 50% overlap. The mean measurement duration in the latter study was 23.4 h [22]. Theoretically, any positive correlation between MABP and r_c_SO_2_ would imply impaired CAR. However, when using surrogate markers for CPP and CBF, both with certain imprecisions, a higher cutoff is usually considered indicative of impaired CAR. Still, a golden standard is lacking [14,16,35,36,37] and the mathematical approach reported by Taylor et al. [34] would not be applicable to our model, as it is based on an analysis in the frequency domain, whereas we used a time-domain approach. We, therefore, based our cutoff of the correlation coefficient between MAPB and r_c_SO_2_ on a literature review, which reported generally used cutoffs ranging between 0.3–0.5 [14]. Secondly, the population studied by Votava-Smith et al. received more inotropes (42% vs. up to 22.7% in our population). This could have affected the %time impaired CAR as it may indicate lower MABP; however, the mean MABP was not reported for comparison [22]. Interestingly, in their study inotropes were not associated with a higher %time impaired CAR. This might have been due to the smaller cohort in their study, possibly keeping the association from reaching statistical significance.

We have several considerations about the association between inotropes and higher %time impaired CAR. Firstly, from our observational study design, it is uncertain whether this was the direct effect of the various inotropes or merely an indicator of low MABP and concomitant CPP. We noticed that infants in our cohort remained to have lower MABP regardless of inotrope administration. This may have caused a CPP outside the autoregulatory plateau. However, Solanki et al. confirmed a dose-dependent effect of dopamine on CAR specifically, despite controlling for illness severity [38]. Secondly, both Solanki and Eriksen et al. propose a rightward shift of the autoregulatory plateau, caused by dopamine-induced sympathetic nervous system activation, as an explanation for impaired CAR [27,38]. Those effects may occur if dopamine crosses the blood–brain barrier, which was reported in preterm neonates [38]. Lastly, in our regression Model 2 including MABP, both inotropes and MABP were independently associated with higher %time impaired CAR. Though with caution, we interpret this as an association with both variables despite the possibility of confounding by MABP. We recognize that it is debatable whether MABP can be included in the model since it is part of our CAR determination. Therefore, we created a separate model without MABP [14], demonstrating inotropes to be independent of MABP associated with more time with impaired CAR, at least during the first day after birth.

We confirmed our hypothesis that the CHD type, in this case, dTGA, affects %time impaired CAR. We hypothesized that this association is due to intrauterine cerebral hypoxemia possibly in combination with brain immaturity [7,8,23]. It would be possible to analyze the %time impaired CAR based on the fractional tissue oxygen extraction (FTOE = (SpO_2_ − r_c_SO_2_)/SpO_2_) in an attempt to correct for hypoxemia; however, we decided to refrain from this step as in our opinion, this would have introduced another insecurity to our surrogate measurements. Moreover, as we are mostly interested in concomitant changes in both parameters, we believe the added value of using FTOE is limited for CAR assessment. Nevertheless, differences in CHD groups should be interpreted with caution as various classifications exist [39,40,41].

We could not confirm a relation between %time impaired CAR and the development of PVE, or mortality. This analysis should be interpreted with caution, as this was a retrospective study and PVE is a rather non-specific sign of cerebral hypoxia.

This study had several strengths. To the best of our knowledge, this is the first study that assessed %time impaired CAR and clinical determinants including the type of CHD as well as outcome in neonates with severe CHD. In one previous study, CAR was assessed in pre-operative CHD neonates [22]; however, the effect on outcomes like cerebral ultrasound examination or mortality was not examined. We included a relatively large number of neonates for analysis. Measuring r_c_SO_2_ with NIRS is standard clinical practice for neonates with CHD in our NICU. This technique is highly feasible due to its non-invasiveness and the possibility of real-time monitoring at the bedside. Choosing r_c_SO_2_ as a surrogate for CBF and the literature-based %time impaired CAR cutoff improved the generalizability of our findings. With our data, we hope to create reference values for CAR in neonates with CHD, a group in which CAR has not been studied extensively so far.

Nevertheless, we recognize the limitations of this study. First, for this retrospective analysis, inclusion depended on available MABP measurements with an indwelling catheter. This may have caused selection bias towards more severely ill neonates. For the same reason, it was not ethical to measure the MABP in healthy age-matched controls. Despite the predominance of dTGA in the CHD population, we did not consider this a case of selection bias, as the number of neonates with dTGA among the excluded neonates was not remarkably lower. Among the 18 excluded dTGA neonates, 6 (33.3%) had an indication for balloon atrial septostomy, of which one did not succeed for technical reasons. Secondly, the various approaches to assessing %time impaired CAR limit the generalizability of CAR determination [35]. Although r_c_SO_2_ and MABP are merely surrogates, we think that they currently provide the best estimates for continuous measurement of CBF and CPP in neonates. Assuming stable cerebral metabolism, we consider changes in r_c_SO_2_ representative of changes in cerebral perfusion. Additionally, manual artifact removal and selection of measurement periods may have introduced unintended selection bias. Thirdly, we assessed signs of ischemia/hypoxia on cerebral ultrasound and mortality, but not long-term neurological functioning. A sensitive measure for this purpose should be added in future studies to better assess the relationship between %time impaired CAR and neurological outcome. Lastly, there was a relatively large number of neonates with a need for intubation and/or sedation in this cohort. This may indicate the illness severity in this population, but sedation itself may also affect cerebral oxygen consumption. We cannot fully exclude a potential confounding effect of sedation in this cohort.

## 5. Conclusions

Neonates with severe CHD have impaired CAR during 9.3% of the 2 h measurement episode on the first day after birth, decreasing to 4.6% on day 3.

Treatment with inotropes, lower MABP, and dTGA were associated with higher %time impaired CAR. Further investigation of the relationship between these determinants and %time impaired CAR is warranted. More research is needed to determine the relationship between %time impaired CAR and neurological outcome in neonates with CHD, using more sensitive outcome measures.

## Figures and Tables

**Table 1:** Clinical characteristics of the study cohort (N = 57).

Clinical Characteristics	N (%) or Mean ± SD or Median (Range)		
CHD group, main defect			
dTGA ± combined defects	24 (42.1)		
Balloon atrial septostomy	14 (58.3)		
Septostomy on day 1	12 (85.7)		
Septostomy on day 2	1 (7.1)		
Septostomy on day 3	1 (7.1)		
Right-sided lesion	16 (28.1)		
Hypoplastic aortic arch/coarctation	12 (21.1)		
Other	5 (8.8)		
Inborn	51 (89.5)		
Male	40 (70.2)		
Gestational age, weeks	38.9 ± 0.8		
Birth weight, grams	3276.5 ± 509.7		
Apgar score at 5 min	8.5 (3–10)		
PPHN	17 (29.8)		
Sepsis	2 (3.5)		
Blood transfusion	5 (8.8)		
Signs of cerebral ischemia/hypoxia	11 (19.3)		
	Day 1 (*n* = 44)	Day 2 (*n* = 54)	Day 3 (*n* = 48)
Prostaglandin E_1_, *n* (%)	41 (93.2)	47 (87.0)	42 (87.5)
Inotropes, *n* (%)	10 (22.7)	13 (24.1)	10 (20.8)
Sedatives, *n* (%)	23 (52.3)	28 (51.9)	21 (43.8)
Diuretics, *n* (%)	0 (0)	5 (9.3)	6 (12.5)
Ventilatory support			
None, *n* (%)	9 (20.5)	16 (29.6)	13 (27.1)
Non-invasive, *n* (%)	14 (31.8)	13 (24.1)	15 (31.3)
Invasive, *n* (%)	21 (47.7)	25 (46.3)	20 (41.7)
	(*n* = 39)	(*n* = 48)	(*n* = 33)
PCO_2_, kPa	4.7 ± 0.8	5.0 ± 1.4	5.1 ± 0.7
Survival, *n* (%)	50 (87.7)		
Follow-up, days	18 (9–21)		

Data are presented as number (percentage), mean ± standard deviation or median (range). dTGA, dextro-transposition of the great arteries; Right-sided lesion, dysplastic/stenotic/absent tricuspid or pulmonary valve. PPHN, persistent pulmonary hypertension of the newborn. Numbers vary per day since CAR measurements were not available for all neonates on all days in this retrospective study.

**Table 2:** Hemodynamic measures per day. Primary endpoint: %time impaired CAR.

Measured Variable	Day 1 (*n* = 44)	Day 2 (*n* = 54)	Day 3 (*n* = 48)
Time with impaired CAR, %	9.3 (0.0–60.0)	5.7 (0.0–47.6)	4.6 (0.0–66.3)
MABP, mmHg	45.6 ± 6.0	48.0 ± 5.6	48.3 ± 5.9
r_c_SO_2_, %	66.5 ± 11.8	69.6 ± 11.1	72.0 ± 11.1
SpO_2_, %	90.4 (73.3–99.4)	90.5 (71.3–99.7)	91.4 (79.1–100.0)
CAR, correlation coefficient	−0.08 ± 0.21	−0.14 ± 0.18	−0.16 ± 0.16

Data are presented as mean ± standard deviation or median (range). CAR, cerebrovascular autoregulation; MABP, mean arterial blood pressure; r_c_SO_2_, cerebral tissue oxygen saturation. Numbers vary per day since CAR measurements were not available for all neonates on all days in this retrospective study.

## Data Availability

A summary of all data collected is reported in the current manuscript. Upon reasonable request, anonymous original data can be requested by e-mail at e.kooi@umcg.nl.

## Supplementary Materials

The following supporting information can be downloaded at: https://www.mdpi.com/article/10.3390/children9111686/s1, Table S1: Types of CHD; Table S2: Associations between clinical determinants and %time impaired CAR, determined by univariable linear regression analysis; Table S3: Associations between clinical determinants and %time impaired CAR, determined by linear regression analysis; Table S4: Use of inotropes per CHD group per day.

## References

[1] Hoffman J.I., Kaplan S. (2002). The incidence of congenital heart disease. J. Am. Coll. Cardiol..

[2] (2016). Congenital Heart Disease and Adolescence.

[3] van der Bom T., Bouma B.J., Meijboom F.J., Zwinderman A.H., Mulder B.J. (2012). The prevalence of adult congenital heart disease, results from a systematic review and evidence based calculation. Am. Heart J..

[4] Mulder B.J.M. (2012). Epidemiology of adult congenital heart disease: Demographic variations worldwide. Neth. Heart J..

[5] Marino B.S., Lipkin P., Newburger J.W., Peacock G., Gerdes M., Gaynor J.W., Mussatto K.A., Uzark K., Goldberg C.S., Johnsonjr W.H. (2012). Neurodevelopmental Outcomes in Children With Congenital Heart Disease: Evaluation and Management. Circulation.

[6] Mebius M.J., Kooi E.M., Bilardo C.M., Bos A.F. (2017). Brain Injury and Neurodevelopmental Outcome in Congenital Heart Disease: A Systematic Review. Pediatrics.

[7] Berg C., Gembruch O., Geipel A. (2009). Doppler indices of the middle cerebral artery in fetuses with cardiac defects theoretically associated with impaired cerebral oxygen delivery in utero: Is there a brain-sparing effect?. Ultrasound Obstet. Gynecol..

[8] Kaltman J.R., Di H., Tian Z., Rychik J. (2004). Impact of congenital heart disease on cerebrovascular blood flow dynamics in the fetus. Ultrasound Obstet. Gynecol..

[9] Miller S.P., McQuillen P.S., Hamrick S., Xu D., Glidden D.V., Charlton N., Karl T., Azakie A., Ferriero D.M., Barkovich A.J. (2007). Abnormal Brain Development in Newborns with Congenital Heart Disease. N. Engl. J. Med..

[10] Licht D.J., Shera D.M., Clancy R.R., Wernovsky G., Montenegro L.M., Nicolson S.C., Zimmerman R.A., Spray T.L., Gaynor J.W., Vossough A. (2009). Brain maturation is delayed in infants with complex congenital heart defects. J. Thorac. Cardiovasc. Surg..

[11] Cheng H.H., Ferradal S.L., Vyas R., Wigmore D., McDavitt E., Soul J.S., Franceschini M.A., Newburger J.W., Grant P.E. (2019). Abnormalities in cerebral hemodynamics and changes with surgical intervention in neonates with congenital heart disease. J. Thorac. Cardiovasc. Surg..

[12] Spaeder M., Klugman D., Skurow-Todd K., Glass P., Jonas R.A., Donofrio M.T. (2017). Perioperative Near-Infrared Spectroscopy Monitoring in Neonates With Congenital Heart Disease. Pediatr. Crit. Care Med..

[13] Rhee C.J., Da Costa C.S., Austin T., Brady K.M., Czosnyka M., Lee J.K. (2018). Neonatal cerebrovascular autoregulation. Pediatr. Res..

[14] Kooi E.M., Richter A.E. (2020). Cerebral Autoregulation in Sick Infants. Clin. Perinatol..

[15] Ortinau C.M., Anadkat J.S., Smyser C.D., Eghtesady P. (2018). Intraventricular Hemorrhage in Moderate to Severe Congenital Heart Disease. Pediatr. Crit. Care Med..

[16] Kooi E.M.W., Verhagen E.A., Elting J.W.J., Czosnyka M., Austin T., Wong F.Y., Aries M.J.H. (2017). Measuring cerebrovascular autoregulation in preterm infants using near-infrared spectroscopy: An overview of the literature. Expert Rev. Neurother..

[17] Panerai R.B., Dineen N.E., Brodie F.G., Robinson T.G. (2010). Spontaneous fluctuations in cerebral blood flow regulation: Contribution of PaCO_2_. J. Appl. Physiol..

[18] Verhagen E.A., Van Braeckel K.N.J.A., Van Der Veere C.N., Groen H., Dijk P.H., Hulzebos C.V., Bos A.F. (2014). Cerebral oxygenation is associated with neurodevelopmental outcome of preterm children at age 2 to 3 years. Dev. Med. Child Neurol..

[19] Volpe J.J. (2001). Neurobiology of Periventricular Leukomalacia in the Premature Infant. Pediatr. Res..

[20] Wernovsky G., Licht D.J. (2016). Neurodevelopmental Outcomes in Children With Congenital Heart Disease—What Can We Impact?. Pediatr. Crit. Care Med..

[21] Sood B.G., McLaughlin K., Cortez J. (2015). Near-infrared spectroscopy: Applications in neonates. Semin. Fetal Neonatal Med..

[22] Votava-Smith J.K., Statile C.J., Taylor M.D., King E.C., Pratt J.M., Nelson D.P., Michelfelder E.C. (2017). Impaired cerebral autoregulation in preoperative newborn infants with congenital heart disease. J. Thorac. Cardiovasc. Surg..

[23] Mebius M.J., Clur S.A.B., Vink A., Pajkrt E., Kalteren W.S., Kooi E.M.W., Bos A.F., Sarvaas G.J.D.M., Bilardo C.M. (2019). Growth patterns and cerebroplacental hemodynamics in fetuses with congenital heart disease. Ultrasound Obstet. Gynecol..

[24] Wong F.Y., Leung T.S., Austin T., Wilkinson M., Meek J.H., Wyatt J.S., Walker A.M. (2008). Impaired Autoregulation in Preterm Infants Identified by Using Spatially Resolved Spectroscopy. Pediatrics.

[25] da Costa C.S., Czosnyka M., Smielewski P., Mitra S., Stevenson G.N., Austin T. (2015). Monitoring of Cerebrovascular Reactivity for Determination of Optimal Blood Pressure in Preterm Infants. J. Pediatr..

[26] Mitra S., Czosnyka M., Smielewski P., O’Reilly H., Brady K., Austin T. (2014). Heart rate passivity of cerebral tissue oxygenation is associated with predictors of poor outcome in preterm infants. Acta Paediatr..

[27] Eriksen V.R., Hahn G.H., Greisen G. (2014). Dopamine therapy is associated with impaired cerebral autoregulation in preterm infants. Acta Paediatr..

[28] Abecasis F., Dias C., Zakrzewska A., Oliveira V., Czosnyka M. (2021). Monitoring cerebrovascular reactivity in pediatric traumatic brain injury: Comparison of three methods. Child’s Nerv. Syst..

[29] Brady K.M., Lee J.K., Kibler K.K., Smielewski P., Czosnyka M., Easley R.B., Koehler R.C., Shaffner D.H. (2007). Continuous Time-Domain Analysis of Cerebrovascular Autoregulation Using Near-Infrared Spectroscopy. Stroke.

[30] Sorrentino E., Diedler J., Kasprowicz M., Budohoski K.P., Haubrich C., Smielewski P., Outtrim J.G., Manktelow A., Hutchinson P.J., Pickard J.D. (2012). Critical thresholds for cere-brovascular reactivity after traumatic brain injury. Neurocrit. Care.

[31] Verhagen E.A., Hummel L.A., Bos A.F., Kooi E.M. (2014). Near-infrared spectroscopy to detect absence of cerebrovascular autoregulation in preterm infants. Clin. Neurophysiol..

[32] Alderliesten T., Lemmers P.M., Smarius J.J., van de Vosse R.E., Baerts W., van Bel F. (2013). Cerebral Oxygenation, Extraction, and Autoregulation in Very Preterm Infants Who Develop Peri-Intraventricular Hemorrhage. J. Pediatr..

[33] Riera J., Cabañas F., Serrano J.J., Bravo M.C., López-Ortego P., Sánchez L., Madero R., Pellicer A. (2014). New Time-Frequency Method for Cerebral Autoregulation in Newborns: Predictive Capacity for Clinical Outcomes. J. Pediatr..

[34] Taylor J.A., Carr D.L., Myers C.W., Eckberg D.L. (1998). Mechanisms Underlying Very-Low-Frequency RR-Interval Oscillations in Humans. Circulation.

[35] Thewissen L., Caicedo A., Lemmers P., Van Bel F., Van Huffel S., Naulaers G. (2018). Measuring Near-Infrared Spectroscopy Derived Cerebral Autoregulation in Neonates: From Research Tool Toward Bedside Multimodal Monitoring. Front. Pediatr..

[36] Chan B., Butler E., Frost S.A., Chuan A., Aneman A. (2018). Cerebrovascular autoregulation monitoring and patient-centred outcomes after cardiac surgery: A systematic review. Acta Anaesthesiol. Scand..

[37] Depreitere B., Citerio G., Smith M., Adelson P.D., Aries M.J., Bleck T.P., Bouzat P., Chesnut R., De Sloovere V., Diringer M. (2021). Cerebrovascular Autoregulation Monitoring in the Management of Adult Severe Traumatic Brain Injury: A Delphi Consensus of Clinicians. Neurocritical Care.

[38] Solanki N.S., Hoffman S.B. (2020). Association between dopamine and cerebral autoregulation in preterm neonates. Pediatr. Res..

[39] Rohit M., Shrivastava S. (2017). Acyanotic and Cyanotic Congenital Heart Diseases. Indian J. Pediatr..

[40] Altit G., Bhombal S., Tacy T.A., Chock V.Y. (2018). End-Organ Saturation Differences in Early Neonatal Transition for Left- versus Right-Sided Congenital Heart Disease. Neonatology.

[41] Van Der Laan M.E., Mebius M.J., Roofthooft M.T., Bos A.F., Berger R.M., Kooi E.M. (2017). Cerebral and Renal Oxygen Saturation Are Not Compromised in the Presence of Retrograde Blood Flow in either the Ascending or Descending Aorta in Term or Near-Term Infants with Left-Sided Obstructive Lesions. Neonatology.

