# Supplementary material for: Early Cerebrovascular Autoregulation in Neonates with Congenital Heart Disease
Source: Children (Basel). 2022 Nov 3;9(11):1686. doi: 10.3390/children9111686 (PMC9688918; doi:10.3390/children9111686)
Supplement: Supplementary file 1 [file children-09-01686-s001.zip › CARCHD-Suppl_Table S1.pdf]

Supplemental Digital Table S1. Types of CHD

**CHD group**	**CHD type/combination**	**N = 57**
dTGA ± combined defects	dTGA	14 (24.6)
	dTGA + ASD/VSD	6 (10.5)
	dTGA + DORV	1 (1.8)
	dTGA + monoventricle	1 (1.8)
	dTGA + DILV + PA	1 (1.8)
	dTGA + MA + PA + HLH	1 (1.8)
Right-sided	PA	4 (7.0)
	Tetralogy of Fallot + PA	4 (7.0)
	Ebstein anomaly	2 (3.5)
	Ebstein anomaly + pulmonary stenosis	1 (1.8)
	Pulmonary stenosis	1 (1.8)
	TA (HRH) + VSD	1 (1.8)
	TA + PA + VSD	1 (1.8)
	PA + dysplastic tricuspid valve	1 (1.8)
	DORV + VSD + dysplastic tricuspid valve	1 (1.8)
Hypoplastic aortic arch / coarctation	dTGA + DORV + hypoplastic aortic arch	4 (7.0)
	dTGA + DILV + hypoplastic aortic arch	2 (3.5)
	Aortic coarctation + (A)VSD	2 (3.5)
	Hypoplastic aortic arch + VSD	1 (1.8)
	Hypoplastic aortic arch + aortic stenosis	1 (1.8)
	Aortic coarctation + VSD + LVOTO	1 (1.8)
	Aortic coarctation + VSD + hypoplastic aortic arch	1 (1.8)
Other	Aortic stenosis	1 (1.8)
	Mitral stenosis	1 (1.8)
	AVSD + LVOTO + hypoplastic aortic valve	1 (1.8)
	Truncus arteriosus type I	1 (1.8)
	Total anomalous pulmonary venous return	1 (1.8)

Data are presented as number (percentage).

CHD, congenital heart disease; dTGA, dextro-transposition of the great arteries; ASD, atrial septal defect; VSD, ventricular septal defect; DORV, double outlet right ventricle; DILV, double inlet left ventricle; PA, pulmonary atresia; MA, mitral atresia; HLH, hypoplastic left heart; TA, tricuspid atresia; HRH, hypoplastic right heart; LVOTO, left ventricular outflow tract obstruction.
