# Supplementary material for: Early Cerebrovascular Autoregulation in Neonates with Congenital Heart Disease
Source: Children (Basel). 2022 Nov 3;9(11):1686. doi: 10.3390/children9111686 (PMC9688918; doi:10.3390/children9111686)
Supplement: Supplementary file 1 [file children-09-01686-s001.zip › CARCHD_Suppl_Table S2.pdf]

Supplemental Digital Table S2. Associations between clinical determinants and %time impaired CAR, determined by univariable linear regression analysis.

		Univariable analysis		
**Day**	**Variable**	**B [95% CI]**	**P value**	**R^2**
1	PGE_1	7.2 [-10.6-25.1]	0.417	0.02
	Inotropes	23.0 [14.9-31.1]	< 0.001**	0.44
	Sedatives	14.0 [6.0-21.9]	0.001*	0.23
	Diuretics	-	-	-
	GA	0.1 [-6.4-6.6]	0.983	0.00
	BW	0.0 [0.0-0.0]	0.327	0.02
	Apgar	-1.2 [-4.1-1.7]	0.407	0.02
	Ventilation	9.0 [3.9-14.1]	0.001*	0.23
	PCO_2, kPa	-1.2 [-7.8-5.3]	0.702	0.00
	MAB, mmHg	-1.2 [-1.8- -0.5]	0.001*	0.22
2	PGE_1	-4.3 [-12.3-3.8]	0.296	0.02
	Inotropes	5.6 [-0.7-11.8]	0.079*	0.06
	Sedatives	0.1 [-5.3-5.6]	0.958	0.00
	Diuretics	-3.9 [-13.4-5.5]	0.404	0.01
	GA	-0.7 [-4.0-2.7]	0.693	0.00
	BW	0.0 [0.0-0.0]	0.127	0.04
	Apgar	-0.7 [-2.6-1.2]	0.453	0.01
	Ventilation	1.9 [-1.3-5.1]	0.231	0.03
	PCO_2, kPa	0.7 [-1.5-2.8]	0.527	0.01
	MABP, mmHg	-0.5 [-1.0-0.0]	0.046*	0.08
3	PGE_1	2.6 [-7.6-12.7]	0.614	0.01
	Inotropes	9.4 [1.5-17.2]	0.020*	0.11
	Sedatives	4.2 [-2.5-10.9]	0.212	0.03
	Diuretics	-4.7 [-14.8-5.4]	0.355	0.02
	GA	-2.2 [-6.2-1.8]	0.267	0.03
	BW	0.0 [0.0-0.0]	0.869	0.00
	Apgar	0.4 [-1.9-2.6]	0.754	0.00
	Ventilation	3.2 [-0.8-7.3]	0.112	0.05
	PCO_2, kPa	3.1 [-0.3-6.4]	0.072*	0.10
	MABP, mmHg	0.1 [-0.5-0.7]	0.743	0.00

B, unstandardized coefficient; CI, confidence interval; PGE_1, prostaglandin E_1; GA, gestational age; BW, birth weight; MABP, mean arterial blood pressure. * p-value < 0.05; ** p-value < 0.001
