# Supplementary material for: Early Cerebrovascular Autoregulation in Neonates with Congenital Heart Disease
Source: Children (Basel). 2022 Nov 3;9(11):1686. doi: 10.3390/children9111686 (PMC9688918; doi:10.3390/children9111686)
Supplement: Supplementary file 1 [file children-09-01686-s001.zip › CARCHD_Suppl_Table S3.pdf]

Supplemental Digital Table S3. Associations between clinical determinants and %time impaired CAR, determined by linear regression analysis

		**Univariable analysis**			**Multivariable model 1**			**Multivariable model 2**		
**Day**	**Variable**	**B [95% CI]**	**P value**	**R^2**	**B [95% CI]**	**P value**	**R^2**	**B [95% CI]**	**P value**	**R^2**
1	Inotropes	23.0 [14.9-31.1]	< 0.001**	0.44	20.5 [11.3-29.6]	< 0.001**	0.51	19.5 [10.6-28.3]	< 0.001**	0.56
	Sedatives	14.0 [6.0-21.9]	0.001*	0.23	-3.1 [-15.0-8.9]	0.607	0.51	-4.4 [-16.0-7.1]	0.441	0.56
	Ventilation	9.0 [3.9-14.1]	0.001*	0.23	6.5 [-0.5-13.5]	0.069	0.51	5.5 [-1.3-12.3]	0.111	0.56
	MABP (mmHg)	-1.2 [-1.8- -0.5]	0.001*	0.22	Not entered	Not entered	Not entered	-0.6 [-1.2-0.0]	0.047*	0.56
2	Inotropes	5.6 [-0.7-11.8]	0.079*	0.06	5.6 [-0.7-11.8]	0.079	0.06	4.2 [-2.1-10.6]	0.186	0.11
	MABP (mmHg)	-0.5 [-1.0-0.0]	0.046*	0.08	Not entered	Not entered	Not entered	-0.5 [-0.9-0.1]	0.103	0.11
3	Inotropes	9.4 [1.5-17.2]	0.020*	0.11	11.5 [7.1-16.0]	< 0.001**	0.53	11.5 [7.1-16.0]	< 0.001**	0.53
	PCO_2 (kPa)	3.1 [-0.3-6.4]	0.072*	0.10	0.5 [-2.1-3.2]	0.689	0.53	0.5 [-2.1-3.2]	0.689	0.53

B, unstandardized coefficient; CI, confidence interval; MABP, mean arterial blood pressure. * p-value < 0.05; ** p-value < 0.001; for univariable variables see Supplemental Digital Table 2.
