# Supplementary material for: Early Cerebrovascular Autoregulation in Neonates with Congenital Heart Disease
Source: Children (Basel). 2022 Nov 3;9(11):1686. doi: 10.3390/children9111686 (PMC9688918; doi:10.3390/children9111686)
Supplement: Supplementary file 1 [file children-09-01686-s001.zip › CARCHD_Suppl_Table S4.pdf]

Supplemental Digital Table S4. Use of inotropes per CHD group per day

CHD group	Day 1 (n = 44)	Day 2 (n = 54)	Day 3 (n = 48)
dTGA ± combined defects	22	23	22
inotropes	8 (30.8%)	8 (28.6%)	8 (30.8%)
Right-sided lesion	12	16	14
inotropes	1 (8.3%)	2 (12.5%)	1 (7.1%)
Hypoplastic aortic arch /coarctation	8	10	9
inotropes	1 (12.5%)	1 (10.0%)	1 (11.1%)
Other	2	5	3
inotropes	0 (0.0%)	2 (40.0%)	0 (0.0%)

Data are presented as number (percentage). CHD, congenital heart disease; dTGA, dextro-transposition of the great arteries. Numbers vary per day, since CAR measurements were not available for all neonates on all days in this retrospective study.
